# Supplementary material for: Intercalated Poly (2-acrylamido-2-methyl-1-propanesulfonic Acid) into Sulfonated Poly (1,4-phenylene ether-ether-sulfone) Based Proton Exchange Membrane: Improved Ionic Conductivity
Source: Molecules. 2020 Dec 31;26(1):161. doi: 10.3390/molecules26010161 (PMC7795994; doi:10.3390/molecules26010161)
Supplement: Supplementary file 1 [file molecules-26-00161-s001.pdf]

Supplementary material

Intercalated Poly (2-acrylamido-2-methyl-1-propanesulfonic acid) into Sulfonated poly (1,4-phenylene ether-ether-sulfone) based Proton Exchange Membrane: Improved Ionic Conductivity

Murli Manohar, Prem P. Sharma and Dukjoon Kim*

School of Chemical Engineering, Sungkyunkwan University, Suwon, Gyeonggi 440-746, Korea; madhavcsmcri87@gmail.com (M.M.); premsharma15@gmail.com (P.P.S.)

* Correspondence: djkim@skku.edu; Tel.: +82-31-290-7250; Fax: +82-31-290-7270

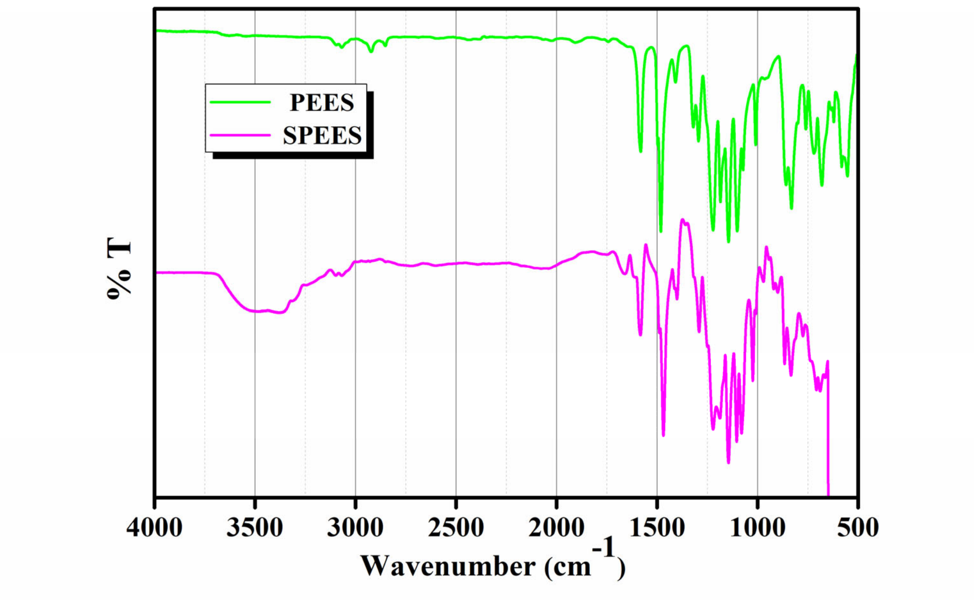

Figure S1. FT-IR spectra of poly (1,4-phenylene ether-ether-sulfone) PEES and sulfonated poly (1,4-phenylene ether-ether-sulfone)

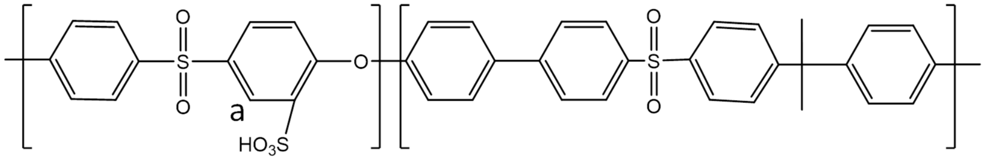

SPEES

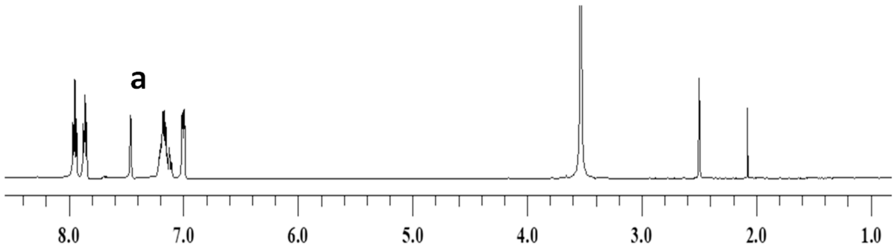

Figure.S2. ^{1}H-NMR spectra of SPEES.
